# Sex hormone-binding globulin may explain sex differences for glucose homeostasis and incidence of type 2 diabetes: the KORA study

**DOI:** 10.1007/s10654-024-01136-2

**Published:** 2024-07-02

**Authors:** Hamidreza Raeisi-Dehkordi, Mojgan Amiri, Wolfgang Rathmann, Tanja Zeller, Jerzy Adamski, Arjola Bano, Yvonne T. van der Schouw, Barbara Thorand, Taulant Muka, Jana Nano

**Affiliations:** 1 Department of Global Public Health and Bioethics, Julius Center for Health Sciences and Primary Care, University Medical Center (UMC) Utrecht, Utrecht University, Utrecht, The Netherlands; 2 Institute of Social and Preventive Medicine (ISPM), University of Bern, Bern, Switzerland; 3 Department of Epidemiology, Erasmus MC University Medical Center, Rotterdam, The Netherlands; 4 Institute for Biometry and Epidemiology, German Diabetes Centre, Leibniz Centre for Diabetes Research at Heinrich Heine University of Düsseldorf, Düsseldorf, Germany; 5 Department of General and Interventional Cardiology, University Heart Center Hamburg, Hamburg, Germany; 6 German Center for Cardiovascular Research (DZHK), Partner Site Hamburg/Kiel/Lübeck, Lübeck, Germany; 7 Institute of Experimental Genetics, Helmholtz Zentrum München, German Research Center for Environmental Health, Ingolstädter Landstraße 1, 85764 Neuherberg, Germany; 8 Department of Biochemistry, Yong Loo Lin School of Medicine, National University of Singapore, 8 Medical Drive, Singapore, 117597 Singapore; 9 Institute of Biochemistry, Faculty of Medicine, University of Ljubljana, Vrazov trg 2, Ljubljana, 1000 Slovenia; 10 Department of Cardiology Inselspital, Bern University Hospital, University of Bern, Bern, Switzerland; 11 Institute of Epidemiology, Helmholtz Zentrum München - German Research Center for Environmental Health, Ingolstädter Landstraße 1, D-85764 Neuherberg, Germany; 12 Partner site Munich-Neuherberg, German Center for Diabetes Research (DZD), Ingolstädter Landstraße 1, D-85764 Neuherberg, Germany; 13 Institute for Medical Information Processing, Biometry and Epidemiology (IBE), Faculty of Medicine, LMU Munich, Pettenkofer School of Public Health, Munich, Germany; 14 Meta-Research Innovation Center at Stanford (METRICS), Stanford University, Stanford 8, CA USA; 15 Epistudia, Bern, Switzerland

**Keywords:** Sex differences, Sex hormone-binding globulin, Glycemic control, Type 2 diabetes, Mediation analysis

## Abstract

Research has indicated that sex hormone-binding globulin (SHBG) is associated with glucose homeostasis and may play a role in the etiology of type 2 diabetes (T2D). While it is unclear whether SHBG may mediate sex differences in glucose control and subsequently, incidence of T2D. We used observational data from the German population-based KORA F4 study (*n* = 1937, mean age: 54 years, 41% women) and its follow-up examination KORA FF4 (median follow-up 6.5 years, *n* = 1387). T2D was initially assessed by self-report and validated by contacting the physicians and/ or reviewing the medical charts. Mediation analyses were performed to assess the role of SHBG in mediating the association between sex (women vs. men) and glucose- and insulin-related traits (cross-sectional analysis) and incidence of T2D (longitudinal analysis). After adjustment for confounders, (model 1: adjusted for age; model 2: model 1 + smoking + alcohol consumption + physical activity), women had lower fasting glucose levels compared to men (β = -4.94 (mg/dl), 95% CI: -5.77, -4.11). SHBG levels were significantly higher in women than in men (β = 0.47 (nmol/l), 95% CI:0.42, 0.51). Serum SHBG may mediate the association between sex and fasting glucose levels with a proportion mediated (PM) of 30% (CI: 22–41%). Also, a potential mediatory role of SHBG was observed for sex differences in incidence of T2D (PM = 95% and 63% in models 1 and 2, respectively). Our novel findings suggest that SHBG may partially explain sex-differences in glucose control and T2D incidence.

**Supplementary Information:**

The online version contains supplementary material available at 10.1007/s10654-024-01136-2.

## Introduction

Ample evidence indicates that the alarming rise in obesity will continue to drive a devastating increase in the prevalence and burden of type 2 diabetes (T2D) over the next decades [1]. The prevalence and incidence of T2D and the associated risk factors such as obesity, glucose and insulin impairments differ according to the sex [2]. In general, prevalence of diabetes is higher in men than women while aging reduces this difference [3]. There is a growing body of evidence that sex differences exist in diabetes outcomes and related complications, highlighting the need for further sex-specific research [4].

Certainly, some of these sex differences are determined by genetics, socio-cultural and most largely by biological factors [5]. Sex hormones and sex hormone binding globulin (SHBG) have been suggested as potential determinants of these sex differences [6]. The association of sex hormones (testosterone, estradiol, etc.) with T2D have been widely addressed [7], however, the independent role of SHBG is still a topic of debate. While SHBG has traditionally been viewed as a passively binding protein that simply regulates the levels of free sex hormones, in recent years its independent biological properties have been highlighted [5]. Emerging evidence suggests that SHBG may directly influence various physiological processes and disease states, offering a promising avenue for further investigation and potential therapeutic development [8]. Typically, men have lower levels of SHBG compared to women [5]. In adult men, SHBG levels are stable for many years but tend to increase with age [9] while SHBG progressively decreases in adult women between the ages of 20 and 60 and then begins to increase [10].

Findings from a large systematic review and meta-analysis of observational studies indicated that lower levels of SHBG are associated with insulin resistance and higher risk of T2D, with stronger associations seen in women compared to men [7]. Mendelian randomization studies have supported a causal role of SHBG in T2D, although the causal effects have been shown to be weaker than the estimates observed in observational studies [11, 12]. However, there is limited evidence on sex-specific mendelian randomization studies on causal role of SHBG in T2D [13]. Therefore, our study lays the groundwork for future research to explore the potentially distinct causal pathways in men and women.

We hypothesized that SHBG levels may explain sex differences for glucose homeostasis and incidence of T2D. To test this hypothesis, we aimed to (i) investigate the association of sex with glucose- and insulin-related traits and incidence of T2D; (ii) to investigate the associations of sex with SHBG and SHBG with glucose- and insulin-related traits and incidence of T2D; and (iii) to assess the potential mediating role of SHBG and its extent in the association of sex with incidence of T2D in a population-based setting of middle-aged and elderly adults. Particularly, we also focused to identify and quantify SHBG’s potential mediating role in broader aspects of glucose homeostasis, such as glucose- and insulin-related traits, an area that has received less attention.

## Methods

### Setting and study population

This study was conducted among participants of the prospective population-based Cooperative Health Research in the Region of Augsburg (KORA) study, selected from population registries in the city of Augsburg (Germany) and two surrounding counties. A total of 4261 middle-aged and older adults, aged 25–74 years, were included at baseline between 1999 and 2001 (KORA S4). Follow-up examinations were performed after 7 years, between 2006 and 2008 (KORA F4) and after 14 years, between 2013 and 2014 (KORA FF4). The KORA F4 study enrolled 3080 participants of whom 2161 were followed up in KORA FF4. All study participants have provided written informed consent. The study was approved by the Ethics Committees of the Bavarian Chamber of Physicians (Ethical Approval Number 06068) adhering to the declaration of Helsinki. Details of the study population and data collection have been reported elsewhere [14].

**Fig. 1:**
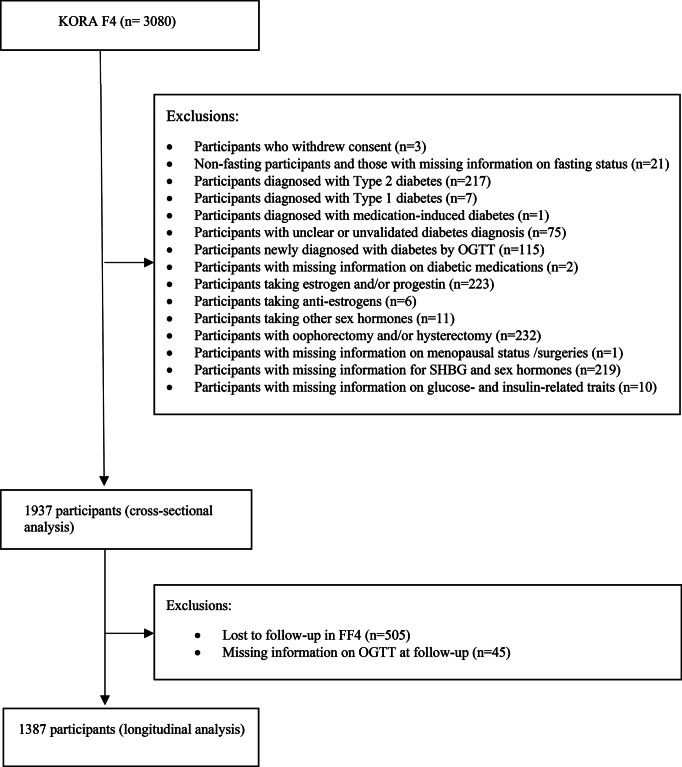
Flowchart for the selection of study participants

The present analysis includes data from the KORA F4 at baseline and KORA FF4 at follow-up. We excluded participants who withdrew consent (*n* = 3), non-fasting participants and participants with missing information on fasting status (*n* = 21), participants with T2D (*n* = 217) and type 1 diabetes (*n* = 7), participants with unclear diabetes diagnosis (*n* = 75), participants newly diagnosed with diabetes by oral glucose tolerance test (OGTT) (*n* = 115), participants diagnosed with medication induced diabetes (*n* = 1), participants with missing information on diabetic medications (*n* = 2), participants taking external hormone therapy (including estrogen and/or progestin, anti-estrogens) (*n* = 240), participants with surgeries (including hysterectomy, oophorectomy) (*n* = 232), participants with missing information on surgeries (*n* = 1), and participants with missing information on SHBG, sex hormones and glucose- and insulin-related traits (fasting glucose levels, insulin levels, 2 h-glucose levels, homeostatic model assessment for insulin resistance (HOMA-IR)) (*n* = 229). Thus, 1937 individuals were included in the cross-sectional analysis, while 1387 participants were included for the longitudinal analysis. (Fig. 1).

### Sexual hormone-binding globulin, glucose homeostasis and T2D assessments

SHBG was measured in serum that was stored at -80 °C, until being assayed. Measurements of SHBG in serum were performed using the ARCHITECT SHBG assay, a chemiluminescent microparticle immunoassay (CMIA) for absolute quantification of SHBG (Abbott Diagnostics) (measuring range SHBG: 0-250 nmol/L, with intra-assay coefficient of variation: 4.29% and inter-assay coefficient of variation range: 6.39–10.3%). Known T2D was self-reported, validated by a physician or medical record review, or self-reported current use of glucose-lowering medications. Participants without known T2D were given a standard 75 g OGTT. Among those receiving an OGTT, newly diagnosed diabetes was defined according to the 1999 World Health Organization diagnostic criteria (i.e. fasting glucose > 6.9 mmol/L and/or 2 h-glucose > 11.0 mmol/L) [15]. Incident diabetes at follow-up was a combination of diabetes clinically diagnosed during the follow-up period plus those who had newly diagnosed diabetes based on OGTT data at FF4 among those who did not have diabetes at baseline. Fasting glucose was measured in fresh serum using hexokinase-G6PD (GLUFlex; Dade Behring, USA). Fasting insulin was measured in thawed serum by an elctrochemiluminescence immunoassay (Cobas e602 Immunoassay Analyser; Roche Diagnostics GmbH, Germany).

### Assessment of covariates

Information on age, sex, medication use (antihypertensive medications and lipid-lowering medications (yes/ no)), hypertension (yes/no), smoking status (regular smoker, irregular smoker, ex-smoker, never-smoker), alcohol consumption (g/day), physical activity (inactive/ active) was collected by trained medical staff using a standardized interview [16]. Body mass index (BMI) was calculated as weight (kg) divided by height squared (m^2^). Waist circumference (cm) was measured at the level midway between the lower rib margin and the iliac crest while the participants breathed out gently. High C-reactive protein was quantified in plasma using a high-sensitivity latex-enhanced nephelometric assay (BN II Analyzer, Dade Behring). Thyroid-stimulating hormone was measured using electrochemiluminescent methods (Dimension Vista Systems; Siemens, Germany). Total cholesterol was measured in fresh serum by enzymatic methods (CHOL Flex).

### Statistical analyses

Continuous data are presented as mean ± SD or as median (inter quartile range (IQR)) when the variables are non-normally distributed. Categorical data are shown as percentages. We log-transformed non-normally distributed variables prior to further regression and subsequently mediation analysis. Differences in baseline characteristics of men and women were assessed with independent-sample t-test or Mann-Whitney U-test for continuous variables and the chi-squared test for categorical variables.

Multivariable linear regression models were used to investigate the association of sex (women vs. men) with SHBG (exposure-mediator) and glucose- and insulin-related traits (fasting glucose levels, 2 h-glucose levels, fasting insulin levels, and HOMA-IR) (exposure-outcome), as well as SHBG with glucose- and insulin-related traits (mediator-outcome). In addition, the association of sex and SHBG with incidence of T2D was assessed using multivariable logistic regression analysis. In model 1, all analyses were adjusted for age and additionally for physical activity, alcohol consumption and smoking in model 2. These models were selected based on potential confounders according to the published literature and the second one was considered as the main model in the mediation analysis.

We performed the mediation analysis to determine whether SHBG is a potential mediator in the association of sex (women vs. men) with glucose- and insulin-related traits and incidence of T2D and if so to what extent. The hypothesized causal structure of the association between sex (the exposure) and the outcome (glucose- and insulin-related traits / incidence of T2D) with SHBG as a mediator are shown as a Directed Acyclic Graph (DAG) in Supplementary Figs. 1–3.

1. We performed several sensitivity analyses to investigate the robustness of our results. 1) We defined a third model with additional adjustments for waist circumference, systolic blood pressure, total cholesterol, high C-reactive protein, thyroid-stimulating hormone, antihypertensive medications and lipid lowering medications. We did not consider model 3 as one of our main models since the covariates in this model are more likely to play an intermediate role in the pathways of investigated associations, rather than being confounders. Thus, the findings of the third model should be interpreted with caution as stated by VanderWeele [17, 18] - the confounding assumptions for mediation analysis are extremely important and violations in these assumptions can give rise to misleading results.
2. To investigate the effect of age, we divided the participants into two groups based on the median age of 53 and repeated the analyses. 3) To account for the effect of weight, we repeated the mediation analysis for subset of individuals with normal weight (BMI < 25) and with overweight and obesity (BMI ≥ 25). 4) To investigate the independent role of SHBG from testosterone, we added testosterone to the main models and repeated the mediation analysis. This sensitivity analysis was run only for fasting glucose levels as an outcome, and we were not able to rerun the analyses for incidence of T2D as the incidence of T2D in the subsamples was limited.

The direct effect (DE), indirect effect (IE), total effect (TE) and proportion mediated (PM) were estimated using regression based approach in a counterfactual framework developed by Valeri and VanderWeele [19]. Non-parametric bootstrapping (200 times) was used to estimate 95% CI and P values. The proportion mediated (%) was estimated as (OR^Direct^ × (OR^Indirect^ – 1)/(OR^Direct^ × OR^Indirect^ – 1) ×100 in the case of a binary outcome (odds ratio(OR)) or as (β^Indirect^/β^Total)^×100 in the case of a continuous outcome (β coefficient (β)) [18]. The DE can be conceived of as the exposure effect on the outcome at a fixed level of the mediator variable, which is different from the TE, the latter representing the overall effect of exposure on the outcome. The IE can be conceived of as the effect on the outcome resulting from the changes of the exposure due to different mediator levels (SHBG). Of note, DE and IE should operate in the same direction in order for the PM to provide meaningful summary [20].

Multiple imputations were done to handle missing values on covariates. Statistical analyses were performed using R statistical software, version 4.2.2 with CMAverse package [21]. All results were considered statistically significant at a p-value < 0.05.

## Results

Baseline characteristics of the overall population and stratified by sex are shown in Table 1. Overall, in the cross-sectional analysis 1937 men (*n* = 1130) and women (*n* = 807) with a mean age of 54.0 ± 12.8 years and median BMI of 26.5 kg/m^2^ (IQR: 24.2, 29.6) were included. The overall mean and SD of fasting glucose and 2 h-glucose were 94.2 ± 9.4 and 106.7 ± 30.1 mg/dl, respectively. Fasting glucose levels, 2 h-glucose levels, fasting insulin levels, and HOMA-IR were significantly higher in women compared to men (*p* < 0.001). No significant difference was observed in the level of physical activity between men and women. Alcohol consumption and intake of medications were higher in men and a sex difference was observed in smoking status (*p* < 0.001). Over a median follow-up of 6.5 years, 99 incident T2D cases (70 men, 29 women) were recorded.

**Table 1:** Baseline characteristics of study participants of KORA F4 in the overall population and stratified by sex

Characteristic	Total sample (*n* = 1937)	Men (*n* = 1130)	Women (*n* = 807)	*p*-value
Age	54.0 ± 12.8	54.8 ± 12.8	52.99 ± 12.7	**0.001**
Body weight (kg)	79.3 ± 15.4	85.4 ± 13.8	70.87 ± 13.5	**< 0.001**
BMI (kg/m^2^)	26.5 (24.2, 29.6)	27.0 (24.86, 29.64)	25.5 (23, 29.55)	**< 0.001**
WC (cm)	93.2 (84.4, 102.1)	97.2 (90.8, 105.2)	84.5 (76.6, 94.5)	**< 0.001**
Fasting glucose levels (mg/dl)	94.2 ± 9.4	96.6 ± 8.9	90.9 ± 9.0	**< 0.001**
2 h-glucose levels (mg/dl)	106.7 ± 30.1	108.2 ± 30.3	104.4 ± 29.7	**0.006**
Fasting insulin levels (µU/ml)	8.5 (6.1, 12)	8.8 (6.5, 12)	7.8 (5.6, 11)	**< 0.001**
HOMA-IR	1.9 (1.3, 2.8)	2.11 (1.5, 3.0)	1.7 (1.2, 2.5)	**< 0.001**
SHBG (nmol/l)	58.1 (40.2, 81)	47.9 (34.9, 64.8)	76.4 (55.9, 101.5)	**< 0.001**
Alcohol consumption (g/day)	8.5 (0.0, 22.8)	16.0 (2.8, 31.4)	2.8 (0.0, 12.3)	**< 0.001**
Physically active n, (%)	1097 (56%)	639 (56%)	458 (57%)	1.00
Smoking				
Regular smoker	331 (17%)	207 (18%)	124 (15%)	**< 0.001**
Irregular smoker	51 (2%)	29 (2%)	22 (2%)	
Ex-smoker	783 (40%)	534 (47%)	249 (30%)	
Never-smoker	769 (39%)	358 (31%)	411 (51%)	
SBP (mmHg)	121.3 ± 18.0	126.4 ± 16.9	114.1 ± 17.0	**< 0.001**
Total cholesterol (mg/dl)	215.5 ± 38.5	215.1 ± 37.8	216.10 ± 39.5	0.58
C-reactive protein (mg/l)	1.0 (0.4, 2.0)	1.0 (0.5, 2.1)	0.9 (0.4, 2.0)	0.29
TSH (mIU/l)	1.2 (0.8, 1.8)	1.2 (0.8, 1.8)	1.2 (0.8, 1.9)	0.61
Use of antihypertensive medications	468 (24%)	300 (26%)	168 (20%)	**0.004**
Use of lipid lowering medications	194 (10%)	131 (11%)	63 (7%)	**0.007**

Data are presented as mean ± SD for normally distributed continuous variables, median (IQR) for non-normally distributed variables or numbers (percentage) for categorical variables. P-values were generated by independent-sample t-test or Mann-Whitney U-test for continuous variables and chi-square test for categorical variables. P-values < 0.05 are shown in bold

*Abbreviations* BMI, Body Mass Index; WC, Waist Circumference; HOMA-IR, Homeostatic Model Assessment for Insulin Resistance; SHBG, Sex Hormone-Binding Globulin; SBP, Systolic Blood Pressure; TSH, Thyroid Stimulating Hormone

### Sex, SHBG, glucose- and insulin-related traits and T2D

Supplementary Table 1 presents the association of sex (women vs. men) with SHBG and glucose- and insulin-related traits. SHBG levels were significantly higher in women than in men. Women had lower fasting glucose, fasting insulin and HOMA-IR levels in both model 1 and 2. No significant sex differences were observed for 2 h-glucose levels.

The association of SHBG and glucose- and insulin-related traits are shown in Supplementary Table 2. Inverse associations of SHBG with fasting and 2 h-glucose levels, fasting insulin, and HOMA-IR were observed in both model 1 and 2.

Sex differences were observed for the incidence of T2D based on our model 2 (OR: 0.56, CI: 0.34, 0.92) (Supplementary Table 3). An inverse association was seen between SHBG and T2D in model 1 (OR: 0.38, CI: 0.23, 0.62) and model 2 (OR: 0.37, CI: 0.22, 0.60) (Supplementary Table 3).

### Mediation analysis

Mediation analysis was performed to assess whether, and to what extent, the sex differences (women vs. men) in glucose- and insulin-related traits and incidence of T2D were mediated by serum SHBG levels.

The results of the mediation analysis on glucose- and insulin-related traits are shown in Table 2. The findings of the mediation analysis showed a significant mediatory effect of SHBG on the association between sex and fasting glucose levels in both DE and IE. DE were (β= -3.68, 95% CI: -4.53, -2.86) in model 1 and (β= -3.42, 95% CI: -4.42, -2.61) in model 2. IE were (β= -1.56, 95% CI: -1.99, -1.18) and (β= -1.51, 95% CI: -1.93, -1.13) in model 1 and 2, respectively. In both models, serum SHBG was estimated to mediate up to PM 30% (CI: 22–41%) of the association.

**Table 2:** Mediation analysis of SHBG on the association between sex (women vs. men [Reference]) and glucose- and insulin-related traits among participants of KORA F4

Effects	ß (95% CI) model 1	*p*-value	ß (95% CI) model 2	*p*-value
Fasting glucose levels				
DE	-3.68 (-4.53, -2.86)	< 0.001	-3.42 (-4.42, -2.61)	< 0.001
IE	-1.56 (-1.99, -1.18)	< 0.001	-1.51 (1.93, -1.13)	< 0.001
TE	-5.24 (-6.02, -4.52)	< 0.001	-4.93 (-5.71, -4.24)	< 0.001
PM	**0.30 (0.22–0.39)**	**< 0.001**	**0.30 (0.22–0.41)**	**< 0.001**
Fasting insulin levels				
DE	0.06 (0.01, 0.12)	0.01	0.03 (-0.01, 0.08)	0.15
IE	-0.17 (-0.20, -0.14)	< 0.001	-0.16 (-0.19, -0.14)	< 0.001
TE	-0.10 (-0.15, -0.06)	< 0.001	-0.13 (-0.17, -0.08)	< 0.001
PM	1.6 (1.12, 2.98)	< 0.001	1.28 (0.91, 1.89)	< 0.001
2 h-glucose levels				
DE	3.33 (0.50, 6.34)	0.05	2.67 (-0.51, 5.28)	0.11
IE	-5.47 (-6.71, -4.18)	< 0.001	-5.17 (-6.84, -3.89)	< 0.001
TE	-2.13 (-4.36, 0.59)	0.08	-2.49 (-5.03, -0.13)	0.04
PM	2.56 (-4.66, 29.69)	0.08	2.07 (0.76, 12.86)	0.04
HOMA-IR				
DE	0.02 (-0.02, 0.07)	0.35	0.00 (-0.04, 0.05)	0.95
IE	-0.18 (-0.22, -0.16)	< 0.001	-0.18 (-0.21, -0.15)	< 0.001
TE	-0.16 (-0.21, -0.11)	< 0.001	-0.18 (-0.24, -0.13)	< 0.001
PM	1.16 (0.86–1.59)	< 0.001	1 (0.78–1.38)	< 0.001

Model 1: Age

Model 2: Model 1 + smoking + alcohol consumption + physical activity

SHBG, fasting insulin levels and HOMA-IR are log-transformed

*Abbreviations* DE, Direct Effect; IE, Indirect Effect; TE, Total Effect; PM, Proportion Mediated

HOMA-IR, Homeostatic Model Assessment for Insulin Resistance

The DE and IE were in opposite directions for mediation analysis of SHBG on the association between sex and 2 h-glucose levels, fasting insulin levels and HOMA-IR (known as inconsistent mediation). In the second model, IE of SHBG for 2 h-glucose levels, fasting insulin levels and HOMA-IR were (β= -5.17, 95% CI: -6.84, -3.89), (β= -0.16, 95% CI: -0.19, -0.14) and (β= -0.18, 95% CI: -0.21, -0.15), respectively.

The mediation analysis of SHBG on the association between sex and incidence of T2D is presented in Table 3. The DE of this association was not statistically significant in neither model 1 (OR: 0.98, 95% CI: 0.61, 1.60) nor model 2 (OR: 0.82, 95% CI: 0.49, 1.28), while the IE was statistically significant in both models 1 (OR: 0.63, 95% CI: 0.50, 0.80) and model 2 (OR: 0.63, 95% CI: 0.50, 0.83), showing the mediatory effect of SHBG on this association. PM was estimated to be 95% in model 1 and 63% in model 2. The results of all sensitivity analyses are provided in Supplementary information 1.

**Table 3:** Mediation analysis of SHBG on the association between sex (women vs. men [Reference]) and incidence of T2D between KORA F4 and FF4.

Effects	OR (95% CI) model 1	*p*-value	OR (95% CI) model 2	*p*-value
DE	0.98 (0.61, 1.60)	0.96	0.82 (0.49, 1.28)	0.47
IE	0.63 (0.50, 0.80)	< 0.001	0.63 (0.50, 0.83)	< 0.001
TE	0.62 (0.42, 0.91)	0.04	0.52 (0.32, 0.81)	< 0.001
PM	**0.95 (0.23, 4.18)**	0.04	**0.63 (0.22, 2.44)**	< 0.001

Model 1: adjusted for age

Model 2: Model 1 + smoking + alcohol consumption + physical activity

SHBG is log-transformed

*Abbreviations* OR, Odds Ratio; SHBG, Sex Hormone-Binding Globulin; T2D, Type 2 Diabetes; DE, Direct Effect; IE, Indirect Effect; TE, Total Effect; PM, Proportion Mediated

## Discussion

To our best knowledge, the current study is the first investigation examining the mediatory role of SHBG, an important biological factor, in explaining sex differences in glucose metabolism and incidence of T2D. Our results indicated a greater level of SHBG in women than in men, which may explain sex differences in glucose levels and incidence of T2D. We observed a high estimated PM by SHBG in the association between sex (women vs. men) and fasting glucose levels (30%) and incidence of T2D (63%).

Our findings on sex differences for SHBG, glucose homeostasis and T2D are in line with previous studies, showing in general higher SHBG levels [22], lower glucose levels [23], lower insulin resistance [23] and lower incidence of T2D [24] in women compared to men.

Our results reinforce previous evidence on the association between SHBG, glucose biomarkers and T2D incidence. The results of a large systematic review and meta-analysis of observational studies on endogenous sex hormones and risk of T2D showed an inverse association of SHBG and risk of T2D in both sexes, although the findings were stronger in women than in men [7]; the same inverse association of SHBG with T2D was found in another systematic review and meta-analysis performed exclusively on women [25]. In support of observational evidence, mendelian randomization studies have also found a causal role of SHBG on T2D [12, 13]. Above all, our study underscores the necessity of developing sex-specific mendelian randomization studies.

A novel finding of our study is the potential mediating role of SHBG on glucose homeostasis. To our knowledge, the current study is the first study that identified SHBG as a potential mediator in the association between sex and glucose hemostasis. We found the mediating effect of SHBG, which was independent of confounding factor and some of potential intermediate factors including age and obesity. Findings of a recent study based on a large population-based cohort on aging, cardiovascular risk and SHBG, found a clear sex-specific pattern of SHBG levels with age. These novel findings highlighted the importance of considering the age-related changes in SHBG levels to avoid controversial results [22]. Thus, we tried to perform the analysis for different subsets of individuals based on median age. Of note, the potentially mediatory role of SHBG on sex differences in glucose levels was observed in participants < 53 years of age and > = 53 years, with a lower PM in those aged 53 and over. The median (IQR) of SHBG levels for individuals aged < 53 were 40.8 (29.6–55.2) and 81.9 (61.4-105.5) and for those > = 53 years were 54.3 (40.2–71.2) and 69.7 (50-97.3) for men and women, respectively. The SHBG levels in men increased with aging, while they decreased in women, resulting in the narrower variation of SHBG levels between men and women in adults aged 53 years and over. This has resulted in lower PM (19–22%) in older individuals compared to higher PM (29–30%) in younger adults.

We also stratified the mediation analysis by BMI categories, given the robust evidence linking overweight and obesity to altered SHBG levels [26]. Interestingly, while the mediation effect of SHBG on sex-differences in glucose levels was strongest in individuals with normal weight, it remained significant across both BMI subsets. The lower PM observed in overweight and obese individuals could be attributed to the well-established reduction in SHBG levels associated with increased adiposity [27, 28].

Various mechanisms have been proposed regarding sex differences in risk of T2D, with steroid hormones being the important ones. Research has indicated that SHBG may interfere with the pathogenesis and development of T2D by regulating the biologic effects of sex hormones (testosterone and estrogen) on peripheral tissues (e.g. muscle, fat and liver).

Increased risk of T2D with low SHBG levels may represent the stronger effects and possible interactions of more bioavailable testosterone and estradiol, and thus, explaining the sex-dependent associations of SHBG. Some studies have proposed a sex-dependent association of SHBG with risk of T2D. For instance, the study done by Haffner et al. indicated that SHBG levels, independent of insulin levels, predict the development of T2D in women but not in men [29]. Another observational study found that SHBG was associated with higher risk of T2D in women rather than men [30]. The findings of a recent mediation analysis on the Masstricht study, investigating the mediatory role of SHBG on the association between intrahepatic lipid content (IHL) and T2D showed greater mediatory role of SHBG in women compared to men, with a PM of 17.2% and 50.9% of SHBG on the association of IHL and T2D for men and women, respectively [31].

Rather than interaction with other sex hormones, there is also a strong evidence supporting independent effect of SHBG on T2D [32]. Studies have found several polymorphisms in the SHBG gene to be associated with insulin resistance and T2D, showing that altered SHBG physiology may trigger the pathogenesis of T2D [11–13]. Additionally, it has been shown that SHBG may mediate cell-surface signaling, cellular delivery, and biological action of sex hormones via activation of a specific plasma receptor directly [12, 33]. To test this hypothesis, we repeated the mediation analysis additionally adjusting for testosterone levels in model 1 and 2 and found that the mediatory role of SHBG was still significant and large for sex differences in glucose levels and T2D. In support of some previous literature, our findings suggest that SHBG may play a more significant role in T2D risk rather than previously recognized mechanisms linked to androgens, which warrants further investigations. However, it’s important to exercise caution when interpreting these results, as our mediation analysis model was adjusted for testosterone, a potential mediator, compromising as such the mediation analysis assumptions [17, 18].

Other novel findings of the current study are the inconsistent mediatory role of SHBG on fasting insulin levels, 2 h-glucose levels and HOMA-IR. While we found no significant associations for the DE of sex on these traits, the IE for the above-mentioned outcomes were significant and considerable, which is sometimes called inconsistent mediation [20].

Our findings could provide more insights to implement randomized clinical trials targeting SHBG in women suffering from low levels of SHBG. Although a large number of clinical trials have been investigating the effect of different interventions like dietary interventions [34], medications [35] and hormonal therapy [36] on SHBG levels, to our knowledge, no clinical trial has investigated the effect of SHBG administration on health outcomes.

This current study has several strengths and limitations. This was the first study to report the mediatory role of SHBG on sex differences in glucose metabolism and incidence of T2D. By using data from the KORA study, we were able to investigate the mediatory role of SHBG on the association of sex and glucose homeostasis in a large cohort of individuals both cross-sectionally and longitudinally with a limited proportion of missing values for confounders. OGTT were performed at both baseline and follow-up which helped us to explore the development of clinically diagnosed T2D and also newly OGTT-diagnosed T2D. Having detailed data on menopausal status (e.g. experiencing oophorectomy or hysterectomy) and on hormonal therapy (e.g. anti-estrogens, estrogens, progestin, hormone replacement therapy, androgens, antiandrogens, enzyme inhibitors, gonadotropin releasing hormones, and anabolic steroids) was helpful to account for these important confounding factors which may not be well-collected in other cohort studies. As a limitation, we were not able to repeat the sensitivity analysis stratified by age and BMI categories for T2D due to the limited number of incident cases. Lack of replication and not considering the non-alcoholic fatty liver disease in our statistical models are other limitations of our study. In addition, we could not perform other preferable modeling such as Cox regression analysis, due to the lack of exact time to event data. Finally, this study was conducted among a German population, which limits the generalizability of our findings to other ethnicities.

In conclusion, in a large-scale population-based study we showed that serum SHBG is a potential mediator in the association between sex and glucose levels as well as incident T2D. Whether SHBG could be a target therapy for reducing sex differences in diabetes needs larger and well-designed clinical studies.

## Electronic supplementary material

Below is the link to the electronic supplementary material.

Supplementary Material 1

## Data Availability

All available data are provided within the manuscript and supplementary files.
