# Supplementary material for: Sex hormone-binding globulin may explain sex differences for glucose homeostasis and incidence of type 2 diabetes: the KORA study
Source: Eur J Epidemiol. 2024 Jul 2;39(8):915–24. doi: 10.1007/s10654-024-01136-2 (PMC11410840; doi:10.1007/s10654-024-01136-2)
Supplement: Supplementary file 1 — Supplementary Material 1 [file 10654_2024_1136_MOESM1_ESM.docx]

***Supplementary materials:*** *Sex hormone-binding globulin may explain sex differences for glucose homeostasis and incidence of type 2 diabetes: The KORA study*

**Results-sensitivity analysis- Supplementary information 1**

We additionally adjusted the association between sex (women vs men) and SHBG and between sex and glucose- and insulin-related traits for other cardiovascular risk factors as model 3. Similar to previous models, women had lower fasting glucose levels and higher SHBG levels than men (**Supplementary Table 4).** We also found an inverse association between SHBG and fasting glucose levels in this model (**Supplementary Table 5**). No association was observed between sex (women vs men) and SHBG with incidence of T2D in the third model (**Supplementary Table 6**). Also, we observed the mediatory role of SHBG on glucose levels while adjusting for further cardiovascular risk factors as well (PM 30%, CI: 22-39%, **Supplementary Table 7**).

We also performed another sensitivity analysis by adding testosterone to model 1 and 2 (**Supplementary Table 8**). PM of SHBG for fasting glucose levels was estimated up to 52% in model 1 and 53% in model 2. Also, PM of SHBG for T2D was up to 36% in model 1 and 31% in model 2.

Moreover, we observed the potential mediatory role of SHBG on the sex differences in fasting glucose levels in both age categories based on median of age (**Supplementary Table 9**). For individuals younger than 53 years old, the PM was 29% (CI:17-40%) in model 1 and the PM 30% (CI:19-43%) in model 2, whereas for those aged 53 and older, the PM was lower (PM 22%, CI:12-36% in model 1 and PM 19%, CI:10-34% in model 2).

Additionally, findings of mediation analysis of SHBG on the association between sex (women vs men) and fasting glucose levels in different categories of BMI showed a PM of 28% (CI:12-49%) in model 1 and PM 29% (CI:12-55%) in model 2 in normal weight individuals while the PM by SHBG for fasting glucose levels were 17% (CI:09-28%) in model 1 and 18% (CI:10-31%) in model 2 in overweight and obese individuals (**Supplementary Table 10**).

**Supplementary Table 1.** Association of sex (women vs men [Reference]) with SHBG and glucose- and insulin-related traits among participants of KORA F4.

| Variable | ß (95 % CI) Model 1 | | ß (95 % CI) Model 2 |
| --- | --- | --- | --- |
| SHBG (nmol/l) | 0.47 (0.43, 0.52) | | 0.47 (0.42, 0.51) |
| Glucose- and insulin-related traits |  |  | |
| Fasting glucose levels (mg/dl) | -5.24 (-6.01, -4.47) | | -4.94 (-5.77, -4.11) |
| Fasting insulin levels (µU/ml) | -0.10 (-0.15, -0.06) | | -0.13 (-0.18, -0.08) |
| 2h-glucose levels (mg/dl) | -2.13 (-4.66, 0.39) | | -2.50 (-5.21, 0.19) |
| HOMA-IR | -0.16 (-0.21, -0.11) | | -0.18 (-0.23, -0.13) |

Model 1: Age.

Model 2: Model 1 + smoking + alcohol consumption + physical activity.

Fasting insulin levels, SHBG and HOMA-IR are log-transformed.

Abbreviations: SHBG, sex hormone-binding globulin; HOMA-IR, homeostatic model assessment for insulin resistance.

**Supplementary Table 2.** Association of SHBG with glucose- and insulin-related traits among participants of KORA F4.

| Variable | ß (95 % CI) model 1 | ß (95 % CI) model 2 |
| --- | --- | --- |
| Fasting glucose levels (mg/dl) | -3.25 (-4.08, -2.42) | -3.22 (-4.05, -2.38) |
| Fasting insulin levels (µU/ml) | -0.36 (-0.40, -0.31) | -0.35 (-0.40, -0.31) |
| 2h-glucose levels (mg/dl) | -11.39 (-14.09, -8.69) | -11.04 (-13.75, -8.33) |
| HOMA-IR | -0.39 (-0.44, -0.34) | -0.39 (-0.44, -0.34) |

Model 1: Age.

Model 2: Model 1+ smoking+ alcohol consumption+ physical activity.

Fasting insulin levels, SHBG and HOMA-IR are log-transformed.

Abbreviations: SHBG, sex hormone-binding globulin; HOMA-IR, homeostatic model assessment for insulin resistance.

**Supplementary Table 3.** Association of sex (women vs men [Reference]) and SHBG with

incidence of T2D between KORA F4 and FF4.

| Variable | OR (95% CI) model 1 | OR (95% CI) model 2 |
| --- | --- | --- |
| Sex (women vs men [Reference]) | 0.67 (0.42, 1.05) | 0.56 (0.34, 0.92) |
| SHBG (nmol/l) | 0.38 (0.23, 0.62) | 0.37 (0.22, 0.60) |

Model 1: Age.

Model 2: Model 1+ smoking+ alcohol consumption+ physical activity.

SHBG is log-transformed.

Abbreviations: OR, odds ratio; T2D; type 2 diabetes; SHBG, sex hormone-binding globulin.

**Supplementary Table 4**. Association of sex (women vs men [Reference])

with SHBG and glucose- and insulin-related traits among participants of KORA F4 (model 3).

| Variable | ß (95 % CI) model 3 |
| --- | --- |
| SHBG (nmol/l) | 0.31 (0.27, 0.36) |
| Glucose- and insulin-related traits |  |
| Fasting glucose levels (mg/dl) | -1.68 (-2.62, -0.73) |
| Fasting insulin levels (µU/ml) | 0.14 (0.10, 0.19) |
| 2h-glucose levels (mg/dl) | 7.05 (3.94, 10.15) |
| HOMA-IR | 0.12 (0.07, 0.17) |

Model 3: Model 2 + waist circumference + SBP + antihypertensive

medications + lipid lowering medications + total cholesterol + CRP + TSH.

SHBG, fasting insulin and HOMA-IR are log-transformed.

Abbreviations: SHBG, sex hormone-binding globulin; HOMA-IR,

homeostatic model assessment for insulin resistance.

**Supplementary Table 5.** Association of SHBG with

glucose- and insulin-related traits among participants of KORA F4 (model 3).

| Variable | ß (95 % CI) model 3 |
| --- | --- |
| Fasting glucose levels (mg/dl) | -1.46 (-2.34, -0.59) |
| Fasting insulin levels (µU/ml) | -0.14 (-1.91, -0.10) |
| 2h-glucose levels (mg/dl) | -5.98 (-8.85, -3.12) |
| HOMA-IR | -0.16 (-0.21, -0.11) |

Model 3: Model 2+ waist circumference + SBP + antihypertensive

Medications + lipid lowering medications + total cholesterol + CRP + TSH.

SHBG, fasting insulin levels, and HOMA-IR are log-transformed.

Abbreviations: SHBG, sex hormone-binding globulin; HOMA-IR,

homeostatic model assessment for insulin resistance.

**Supplementary Table 6.** Association of sex (women vs men [Reference])

and SHBG with incidence of T2D between KORA F4 and FF4. (model 3).

| Variable | OR (95% CI) model 3 |
| --- | --- |
| Sex (women vs men [Reference]) | 0.92 (0.51, 1.63) |
| SHBG (nmol/l) | 0.61 (0.35, 1.04) |

Model 3: Model 2+waist circumference+ SBP+ antihypertensive

medications+ lipid lowering medications+ total cholesterol + CRP+ TSH.

SHBG is log-transformed.

Abbreviations: OR, odds ratio; T2D; type 2 diabetes; SHBG, sex hormone-binding globulin;

SBP, systolic blood pressure; TSH, thyroid stimulating hormone.

**Supplementary Table 7**. Mediation analysis of SHBG on

the association between (women vs men [Reference]) and

glucose- and insulin-related traits (model 3).

| Effects | ß (95% CI) model 3 | p-value |
| --- | --- | --- |
| Fasting glucose levels |  |  |
| DE | -3.68 (-4.53, -2.86) | <0.001 |
| IE | -1.56 (-1.99, -1.18) | <0.001 |
| TE | -5.24 (-6.02, -4.52) | <0.001 |
| PM | **0.30 (0.22-0.39)** | **<0.001** |
| Fasting insulin levels |  |  |
| DE | 0.06 (0.01, 0.12) | 0.01 |
| IE | -0.17 (-0.20, -0.14) | <0.001 |
| TE | -0.10 (-0.15, -0.06) | <0.001 |
| PM | 1.6 (1.12, 2.98) | <0.001 |
| 2h-glucose levels |  |  |
| DE | 3.33 (0.50, 6.34) | 0.05 |
| IE | -5.47 (-6.71, -4.18) | <0.001 |
| TE | -2.13 (-4.36, 0.59) | 0.08 |
| PM | 2.56 (-4.66, 29.69) | 0.08 |
| HOMA-IR |  |  |
| DE | 0.02 (-0.02, 0.07) | 0.35 |
| IE | -0.18 (-0.22, -0.16) | <0.001 |
| TE | -0.16 (-0.21, -0.11) | <0.001 |
| PM | 1.16 (0.86-1.59) | <0.001 |

Model 3: Model 2+ waist circumference + SBP + antihypertensive

medications + lipid lowering medications + total cholesterol + CRP + TSH.

SHBG, fasting insulin levels and HOMA-IR are log-transformed.

Abbreviations:DE, direct effect; IE, indirect effect; TE, total effect; PM, proportion mediated.

**Supplementary Table 8.** Mediation analysis of SHBG on the association between (women vs men [Reference]) and fasting glucose levels and T2D with adding testosterone in the main models.

| Mediation analysis of SHBG and fasting glucose levels | | | | |
| --- | --- | --- | --- | --- |
| Effects | **ß (95% CI) model 1** | **p-value** | **ß (95% CI) model 2** | **p-value** |
| DE | -2.85 ( -5.42, -0.48) | 0.02 | -2.60 (-4.88, -0.45) | 0.03 |
| IE | -3.1 (-4.26, -2.15) | <0.001 | -3.00 (-4.20, -2.12) | <0.001 |
| TE | -5.96 (-8.47, -3.79) | <0.001 | -5.6 (-7.9, -3.78) | <0.001 |
| PM | **0.52 (0.32, 0.88)** | <0.001 | **0.53 (0.32, 0.89)** | <0.001 |
| Mediation analysis of SHBG and T2D | | | | |
| Effects | **OR (95% CI) model 1** | **p-value** | **OR (95% CI) model 2** | **p-value** |
| DE | 0.49 (0.18, 1.2) | 0.15 | 0.43 (0.16, 0.99) | 0.04 |
| IE | 0.45 (0.28, 0.65) | <0.001 | 0.43 (0.27, 0.68) | <0.001 |
| TE | 0.22 (0.08, 0.51) | 0.01 | 0.19 (0.08, 0.48) | 0.01 |
| PM | **0.36 (0.10, 1.17)** | 0.01 | **0.31 (0.08, 0.84)** | 0.01 |

Model 1: Age + testosterone.

Model 2: Model 1+ smoking + alcohol consumption + physical activity + testosterone.

SHBG is log-trasnformed.

Abbreviations: OR, odds ratio; SHBG, sex hormone-binding globulin; T2D, type 2 diabetes; DE, direct effect; IE, indirect effect; TE, total effect; PM, proportion mediated.

**Supplementary Table 9.** Mediation analysis of SHBG on the association between (women vs men [Reference]) and fasting glucose levels stratified by median age (53 years).

| Mediation analysis for individuals < 53 years | | | | |
| --- | --- | --- | --- | --- |
| Effects | **ß (95% CI) model 1** | **p-value** | **ß (95% CI) model 2** | **p-value** |
| DE | -4.44 (-5.46, -3.30) | <0.001 | -4.18 (-5.33, -2.99) | <0.001 |
| IE | -1.82 (2.40, -1.13) | <0.001 | -1.79 (-2.52, -1.10) | <0.001 |
| TE | -6.27 (-7.06, -5.25) | <0.001 | -5.97 (-6.90, -5.01) | <0.001 |
| PM | **0.29 (0.17-0.40)** | **<0.001** | **0.30 (0.19-0.43)** | **<0.001** |
| Mediation analysis for individuals >= 53 years | | | | |
| Effects | **ß (95% CI) model 1** | **p-value** | **ß (95% CI) model 2** | **p-value** |
| DE | -3.28 (-4.45, -1.99) | <0.001 | -3.33 (-4.71, -2.21) | <0.001 |
| IE | -0.93 (-1.31, -0.51) | <0.001 | -0.82 (-1.28, -0.43) | <0.001 |
| TE | -4.22 (-5.40, -3.03) | <0.001 | -4.16 (-5.47, -3.07) | <0.001 |
| PM | **0.22 (0.12-0.36)** | **<0.001** | **0.19 (0.10-0.34)** | **<0.001** |

Model 1: Age.

Model 2: Model 1+ smoking + alcohol consumption + physical activity.

SHBG is log-transformed.

Abbreviations: SHBG, sex hormone-binding globulin; DE, direct effect; IE, indirect effect; TE, total effect; PM, proportion mediated.

**Supplementary Table 10.** Mediation analysis of SHBG on the association between (women vs men [Reference]) and fasting glucose levels stratified by BMI.

| Mediation analysis for individuals with normal body weight (BMI < 25 kg/m^2^) | | | | |
| --- | --- | --- | --- | --- |
| Effects | **ß (95% CI) model 1** | **p-value** | **ß (95% CI) model 2** | **p-value** |
| DE | -3.28 (-4.72, -1.94) | <0.001 | -3.13 (-4.61, -1.55) | <0.001 |
| IE | -1.33 (-2.06, -0.62) | <0.001 | -1.30 (-2.14, -0.50) | <0.001 |
| TE | -4.61 (-5.73, -3.54) | <0.001 | -4.43 (-5.75, -3.28) | <0.001 |
| PM | **0.28 (0.12, 0.49)** | <0.001 | **0.29 (0.12, 0.55)** | <0.001 |
| Mediation analysis for overweight and obese individuals (BMI >= 25 kg/m^2^) | | | | |
| Effects | **ß (95% CI) model 1** | **p-value** | **ß (95% CI) model 2** | **p-value** |
| DE | -3.85 (-4.65, -2.81) | <0.001 | -3.48 (-4.51, -2.40) | <0.001 |
| IE | -0.82 (-1.30, -0.43) | <0.001 | -0.81 (-1.17, -0.44) | <0.001 |
| TE | -4.68 (-5.51, -3.60) | <0.001 | -4.29 (-5.27, -3.22) | <0.001 |
| PM | **0.17 (0.09, 0.28)** | <0.001 | **0.18 (0.10, 0.31)** | <0.001 |

Model 1: adjusted for age.

Model 2: Model 1+ smoking + alcohol consumption + physical activity.

SHBG is log-transformed.

Abbreviations: SHBG, sex hormone-binding globulin; DE, direct effect; IE, indirect effect; TE, total effect; PM, proportion mediated.

**Supplementary Table 11.** Mediation analysis of SHBG on the association between (women vs men [Reference]) and incidence of T2D (model 3).

| Effects | OR (95% CI) model 3 | P value |
| --- | --- | --- |
| NDE | 1.02 (0.59, 1.60) | 0.91 |
| NIE | 0.87 (0.73, 1.05) | 0.15 |
| TE | 0.89 (0.51, 1.50) | 0.69 |
| PM | 1.27 (-8.4, 10.9) | 0.73 |

Model 3: Model 2 + waist circumference + SBP + antihypertensive

medications + lipid lowering medications + total cholesterol + CRP + TSH.

SHBG is log-transformed.

Abbreviations: OR, odds ratio; T2D, type 2 diabetes; SHBG, sex hormone-binding globulin;

DE, direct effect; IE, indirect effect; TE, total effect; PM, proportion mediated.

**
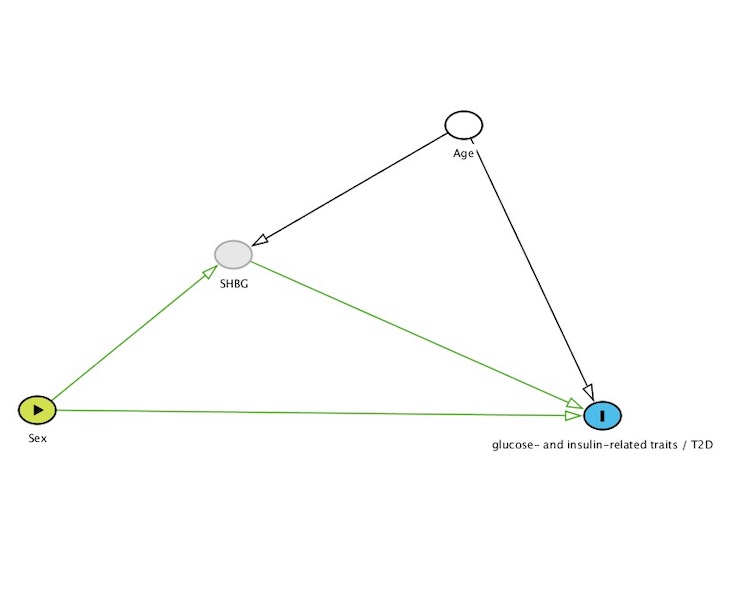
**

**Supplementary Figure 1.** Directed acyclic graph (DAG) for the association of sex through, sex hormone binding globulin (SHBG), on glucose- and insulin-related traits / type 2 diabetes (T2D). Study DAG for model 1, exposure = Sex, mediator = SHBG, outcome = glucose- and insulin-related traits / T2D. for **model 1**, adjustment = age.


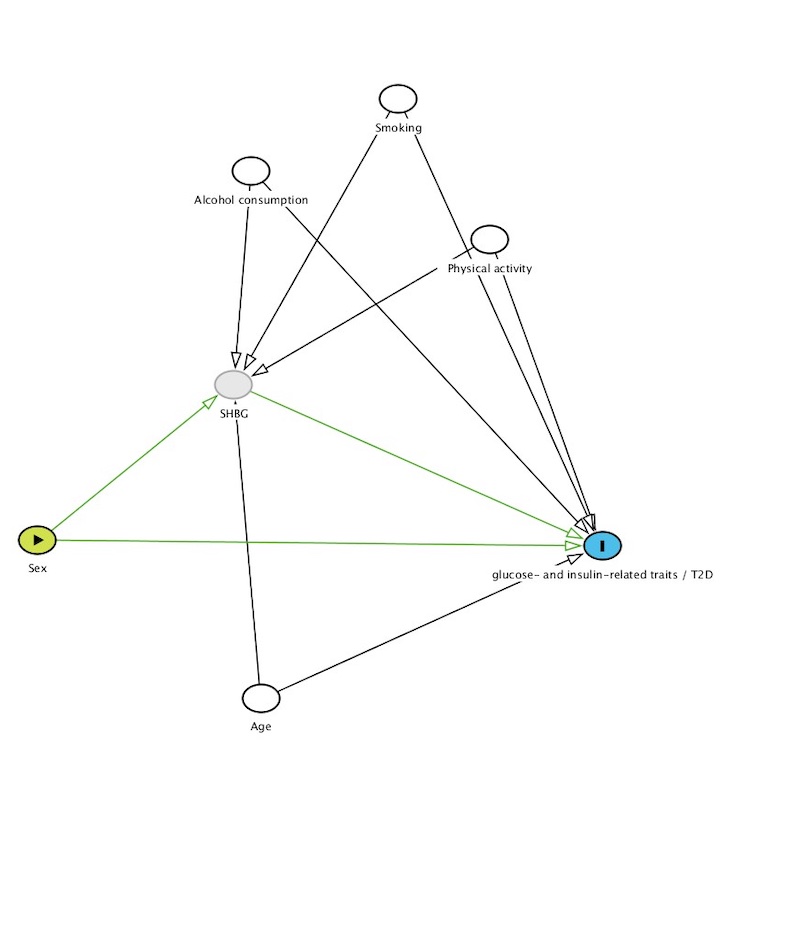


**Supplementary Figure 2.** Directed acyclic graph (DAG) for the association of sex, through sex hormone binding globulin (SHBG), on glucose- and insulin-related traits / type 2 diabetes (T2D). Study DAG for **model 2**, exposure = sex, mediator = SHBG, outcome= glucose- and insulin-related traits / T2D, adjustment variables = age + alcohol consumption + smoking + physical activity.


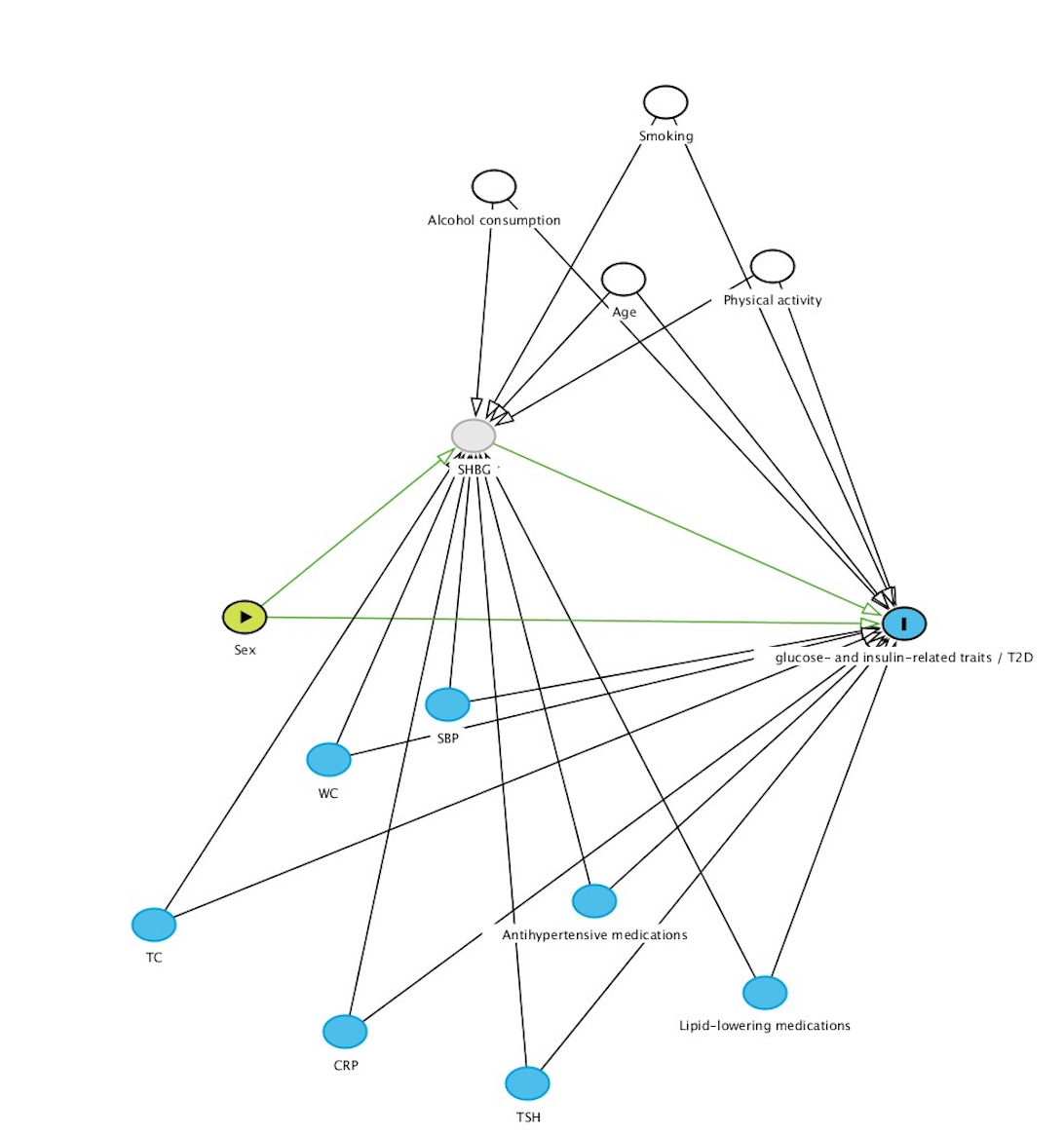


**Supplementary Figure 3**. Directed acyclic graph (DAG) for the association of sex, through sex hormone binding globulin (SHBG), on glucose- and insulin-related traits / type 2 diabetes (T2D). Study DAG for **model 3**, further adjustments for possible mediators (variables in blue). Exposure = Sex, mediator = SHBG, outcome = glucose- and insulin-related traits / T2D, adjustment variables = age + alcohol consumption + smoking, physical activity + waist circumference + SBP + antihypertensive medications+ lipid lowering medications + total cholesterol + CRP+ TSH.
